# Hyponatremia and SARS-CoV-2 infection: A narrative review

**DOI:** 10.1097/MD.0000000000030061

**Published:** 2022-08-12

**Authors:** Elmukhtar Habas, Elrazi Ali, Aml Habas, Amnna Rayani, Hafedh Ghazouani, Fahmi Khan, Khalifa Farfar, Abdel-Nasser Elzouki

**Affiliations:** a Internal Medicine, Hamad Medical Corporation, Doha, Qatar; b Tripoli Children Hospital, Tripoli, Libya; c Quality Department, Hamad Medical Corporation, Doha, Qatar.

**Keywords:** ACE/ACE2, COVID-19, hyponatremia, IL-6, SARS-CoV-2, syndrome of inappropriate antidiuretic hormone

## Abstract

A novel rapid spreading and changing virus called SARS-CoV-2 appeared in Wuhan city in December 2019. It was announced by the World Health Organization (WHO) as a pandemic disease in March 2020. It commonly presents with respiratory symptoms; however, it may be asymptomatic. Electrolyte abnormalities are not uncommon features of SARS-CoV-2 infection. Hyponatremia is one of these electrolyte disturbances among SARS-CoV-2 patients, and it may produce symptoms such as weakness and seizure as the initial presenting symptoms. The underlying mechanism(s) of hyponatremia due to SARS-CoV-2 infection is (are) not established.

The aim of this review is to evaluate the possible mechanism of hyponatremia in patients with COVID-19. Understanding and categorizing the hyponatremia in these patients will lead to better treatment and correction of the hyponatremia.

A review of the literature between December 2019 and March 2022 was conducted searching for the possible reported mechanism(s) of hyponatremia in SARS-CoV-2.

Although SIADH is the commonly reported cause of hyponatremia in SARS-CoV-2 infection, other causes such as diarrhea, vomiting, and kidney salt loss must be considered before SIADH.

## 1. Introduction and Background

Coronavirus disease-2019 is caused by the SARS-CoV-2 virus, leading to severe acute respiratory syndrome. SARS-CoV-2 is a member of the RNA beta-coronavirus family, which includes the virus that causes outbreaks of severe acute respiratory syndrome and Middle East respiratory syndrome during 2012 and 2013.^[1]^ SARS-CoV2 was labeled a pandemic by The World Health Organization (WHO) in March 2020, assembling it one of the most devastating pandemics in human history. There are more than 192 million people infected by SARS-CoV-2 and more than 4 million confirmed deaths worldwide, and the number of newly infected patients and deaths are rising dramatically every day, especially with the new variants of SARS-CoV-2, such as delta-variant.^[2]^

SARS-CoV-2 pneumonia commonly manifests as fever, breathlessness, cough, and chest X-ray infiltrates.^[3]^ Muscle pain, headache, tachypnea, difficulty breathing, sore throat, taste and smell loss, rhinorrhea, and abdominal pain associated with diarrhea and nausea/vomiting are commonly reported presentations in SARS-CoV-2 patients.^[4]^ Other manifestations include thrombotic complications, conjunctivitis, and varied skin lesions such as maculopapular, vesicular eruptions, or transitory livedo reticularis, have been reported in these patients.^[3]^ Recently, a case report showed the activation of stable guttate psoriasis.^[5]^ Acute respiratory distress syndrome (ARDS), respiratory failure, organ dysfunction, and death are all possible outcomes of severe SARS-CoV-2 infection.^[6,7]^ The death rate was higher in patients with previous medical comorbidities.^[8,9]^ In contrast, a significant portion of SARS-CoV-2 individuals may be asymptomatic, making diagnosis more challenging,^[3]^ causing delays in diagnosis and early treatment, increasing the rate of death.

Hyponatremia is the most prevalent electrolyte disturbance associated with a higher rate of death.^[10]^ A sodium level of 135 mmol/L in the serum is the lower cut-off point for normal-natremia.^[11]^ Hyponatremia is induced by different mechanisms in various situations.^[12]^ Hypovolemic, normovolemic, and hypervolemic hyponatremia are the 3 types of sodium disturbance; and each type of hyponatremia generally has a different therapeutic strategy.^[13]^

Body fluid and plasma fluid contents are the main determinants of serum sodium levels. Interestingly, the serum sodium concentration is controlled mainly by the plasma water content rather than the sodium content. Both sodium and water balance are maintained by 2 different physiological mechanisms. Osmoregulation is the first mechanism regulating water excretion and stimulation of thirst centers via antidiarrheic hormone (ADH) secretion via the hypothalamus. The second mechanism is via the effect of the renin-angiotensin-aldosterone system (RAAS) on the distal convoluted tubule.

Inflammatory markers such as interleukin-6 (IL-6) appear to increase in SARS-CoV-2 infection, and its level is directly related to hyponatremia and the rate of mortality.^[14]^ Other electrolyte disturbances have been reported as severe SARS-CoV-2 infections, including hypokalemia and hypocalcemia^[15]^; however, serum sodium significantly affects the outcome of COVID-19 treatment.^[16,17]^

SARS-CoV-2 viral infection can inflict direct kidney injury; additionally, it can downregulate angiotensin-converting enzyme 2 (ACE2) by activating type 1 angiotensin receptor and reducing angiotensin synthesis,^[18]^ which worsens AKI. Chronic kidney disease (CKD) patients, especially diabetic patients with SARS-CoV-2, are at a higher risk of AKI due to baseline upregulation of angiotensin-converting enzyme (ACE) and downregulation of ACE-2 protein. High salt intake has been reported to boost the glomerular ACE/ACE2 ratio, which promotes oxidative stress and progressive kidney damage.^[19]^ All of these possible causes or factors underlying the hyponatremia in SARS-CoV-2 patients, and all of these possible mechanisms will be thoroughly reviewed.

## 2. Method

We looked for relevant research articles in PubMed form December 2019 to March 2022, using the key words hyponatremia combined with COVID-19/SARS-CoV-2. The search included case reports, reviews, and original articles. The search revealed 119 articles which were screened. Articles describing hyponatremia in adults above 18 years were included. Grey literature and nonEnglish articles were excluded.

Ethical approval was not required from the Medical research Centre in Doha, because it is a narrative review of the preexisting published articles.

## 3. Review

### 3.1. COVID-19-associate hyponatremia epidemiology

The exact epidemiology of hyponatremia in SARS-CoV-2 infection has not been reported in certainty; all the reported hyponatraemia cases were either case reports or case series reports. Hyponatremia in SARS-CoV-2 infected cases is caused by different factors.^[12,20]^ During the 2003 SARS infection, hyponatremia was reported in 60% of patients.^[21]^ In the SARS-CoV-2 pandemic, there are no precise data on the rate of hyponatremia in SARS-CoV-2 infection. Recently, Aggarwal et al reported a 50% prevalence of hyponatremia in a small sample of SARS-CoV-2 patients with hyponatremia.^[22]^ Ho et al reported a case of COVID-19 with hyponatremia that initially presented with seizures.^[23]^ A published case series of patients diagnosed with COVID-19 had hyponatremia-related SIADH.^[24]^ In all reported patients in the series, hyponatremia was acute and severe, and hyponatremia was mostly due to SIADH.^[25]^

### 3.2. Mechanisms of COVID-19-associated hyponatremia

Other causes of hyponatremia, such as fluid overload, salt, and fluid loss through the kidney, gastrointestinal, and excessive sweating must be excluded before relating the hyponatremia to the SIADH. This needs a good history, including drug history, especially diuretics, clinical examination, assessment of fluid volume status, and urine electrolyte measurement. Before diagnosing SIADH as a cause of hyponatremia, it is necessary to satisfy the diagnostic criteria for SIADH and rule out other secondary causes of hyponatremia.

#### 3.2.1. Endocrine-mediated mechanism.

The etiology of hyponatremia in SARS-CoV-2 is unclear; an interplay of multiple organs was included. Although it had appeared that the kidney has no share in hyponatremia development in SARS-CoV-2 infection previously, and most of the main mechanism of hyponatremia was SIADH.^[25,26]^ Recently, it was reported that the kidneys are involved in SARS-CoV-2 -associated hyponatremia. Generally, it seems that there are 3 possible mechanisms that may lead to hyponatremia: excess ADH secretion either due to hypothalamic-pituitary-adrenal axis abnormality and/or ectopic ADH production due to SARS-CoV-2 pneumonia, and fluid plus sodium loss.

Vasopressin hormone is produced by the hypothalamic supraoptic and paraventricular nuclei and stores and releases normally from the posterior lobe of the pituitary gland. Vasopressin can also be produced from nonpituitary sources. Excess release of the hormone from the pituitary gland or other sites, and its ongoing impact on vasopressin receptors causes SIADH. In 1967, Schwartz and Bartter reported for the first time a high ADH in 2 lung cancer patients. The essential criteria for SIADH diagnosis are low serum sodium in patients with either hypervolemia or euvolemia with high ADH levels.^[27]^

SIADH was suggested as a cause of hyponatremia in SARS-CoV-2 pneumonia,^[25]^ and in a case initially presented with seizure in SARS-CoV-2 patient with hyponatremia.^[23]^ This proactive desmopressin-based mechanism was supported in these patients who showed drastic improvement following hyponatremia correction by intravenous double strength saline and fluid restriction for 4 days.^[23,25]^ Last year, a published case series of SIADH-induced COVID-19 pneumonia reported that sudden very low serum sodium was noted,^[28]^ and again the hyponatremia improved with fluid restriction.

Another possibility of SIADH-related hyponatremia in SARS-CoV-2 infection is due to a nonsuppressible vasopressin secretion even in the hypotonic state, stimulating kidney water reabsorption, mostly due to the central IL-6 effect, despite the low serum cortisol level and normal RAAS activity.^[29]^

It was noted that the high interleukin-6 level impairs the osmoregulatory mechanism of ADH in the brain, causing hyponatremia.^[30]^ Increased serum IL-6 concentration mediates the release of central vasopressin hormone.^[31]^ It was reported that 17 out of 29 patients with hyponatremia who were confirmed to have SARS-CoV-2 infection, their serum IL-6 level was typically high and inversely related to the serum sodium concentration, whereas the level of serum sodium was directly related to PaO2/FiO_2_.^[14]^

Although there is no strong evidence supporting the impairment of hypothalamic-pituitary stimulation, causing less adrenocorticotropic hormone release by the hypothalamus and pituitary adrenocorticotropic hormone (ACTH) secretion. Low ATCH impairs cortisol formation and its release from the suprarenal gland in SARS-CoV-2 infected patients. There was a reported case of hyponatremia and pituitary macroadenoma in a SARS-CoV-2 -infected patient, and the hyponatremia was unexplainable by either thiazide diuretics or vomiting,^[32]^ and SIADH was thought to be the underlying cause of hyponatremia.

**Figure 1.**
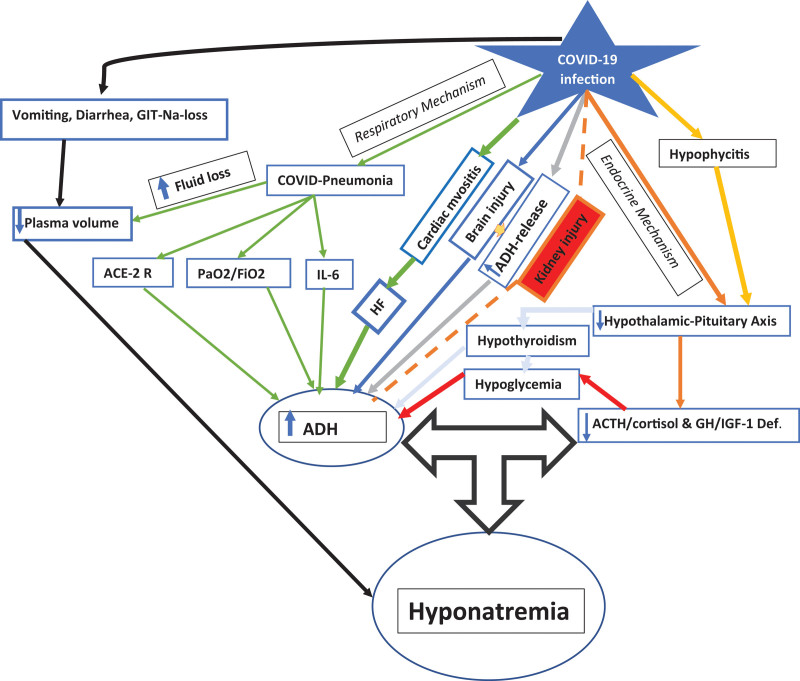
The figure shows the pathogenesis of hyponatremia in COVID 19 patient.

Forty percent of 61 SARS-CoV-2 patients were followed after recovery; they showed evidence of centrally induced hypocortisolism.^[33]^ This central hypocortisolism guided the authors to suggest the probability of coronavirus infection-induced reversible hypophysitis, or direct hypothalamic damage by the SARS virus, causing hypothalamic and/or pituitary malfunction.^[34]^ Other factors that may cause SARS-CoV-2 -induced hyponatremia include secondary hypothyroidism, which can occur following SARS-CoV-2 hypophysitis (Fig. 1). Moreover, SARS-CoV-2 infection can causes stress which can cause hyperglycemia and cause functional hyponatremia.^[35]^

#### 3.2.2. Kidney damage.

Kidney damage can be caused by hemodynamic disturbances and/or insufficient immunological response to SARS-CoV-2 infection.^[13]^ According to a study that showed the presence of viral particles in the proximal convoluted tubule (PCT) and podocytes indicates, to some extent, direct renal cell damage by SARS-CoV-2 virus infection.^[36]^ Damage to the kidneys by SARS-CoV-2 infection usually causes proteinuria and hematuria, and sometimes, acute kidney impairment and failure. Moreover, acute inflammatory cytokine (IL-6) release in COVID-19 patients is known to precipitate acute kidney injury (AKI), glomerular pathology,^[37]^ acute tubular necrosis, leading to PCT damage,^[36]^ and electrolyte abnormalities.

The angiotensin-converting enzyme 2 (ACE2), a receptor that mediates SARS-CoV-2 entry into cells, is extensively expressed in the PCT and glomeruli cells, increasing the incidence of kidney injury in SARS-CoV-2 infection. ACE2 expresses receptors and enzymes that viruses employ as gateways.^[24,36]^ ACE2 enzymes and receptors are also present in the lungs, heart muscles, and bowel, causing damage to these organs in COVID-19 patients.^[14]^

Another mechanism that may be involved in the renal impairment of SARS-CoV-2 is cytokine-mediated inflammation. It is recognized that cytokine cascade activation can lead to several renal pathological changes, such as tubular necrosis, PCT malfunction, glomerulopathy, acute renal function impairment, and electrolyte derangement.^[38]^

Specific features of PCT malfunction in individuals with COVID-19 have been reported. A cohort study showed evidence of PCT dysfunction,^[38]^ manifesting as low molecular proteinuria (70 %–80%), aminoaciduria (46%), and mishandling of uric acid (46%) and/or phosphate in 19% of the enrolled patients. Selective damage to some function of PCT is evident in those enrolled patients who had no glucosuria when their blood sugar was normal, despite the occurrence of other features of PCT damage.^[38]^ The kidney histopathology of infected SARS-CoV-2 patients showed clear PCT injuries, loss of the brush border with a marked reduction in megalin expression, and intraluminal debris deposition. Coronaviruse-like particles were detected in the endoplasmic reticulum vacuoles and cisternae of PCT in these patients. Interestingly, hypouricemia and uricosuria were independently linked with disease severity, and these patients required mechanical ventilation because of the associated high risk of respiratory failure reported by the same study. SARS-CoV-2 appears to cause distinct manifestations due to abnormal PCT function, which may provide novel insights into COVID-19 severity and outcome.^[36]^ Hence, both direct and indirect PCT damage by SARS-CoV-2 appears to have a significant role in hyponatremia, which is associated with SARS-CoV-2 infection.

#### 3.2.3. Inflammation-mediated mechanism.

Interleukin-6 (IL-6) is a cytokine produced by monocytes and macrophages that plays a key role in the development of hyponatremia by causing nonosmotic vasopressin release and secondary electrolyte abnormalities and PCT dysfunction.^[38,39]^ The alleviation of hyponatremia with the administration of tocilizumab,^[40]^ which is a humanized monoclonal antibody against the IL-6 receptor, suggesting that IL-6 is implicated in the pathogenesis of hyponatremia-associated SARS-CoV-2 infection.^[14,28]^ Volume depletion accompanying SARS-CoV-2 infection due to excessive fluid loss may also lead to an increase in ADH release. This causes water retention and increases hyponatremia.

Although ARDS is the major cause of mortality in COVID-19 patients, the intense inflammation that occurs with SARS-CoV-2 infection is characterized by increased cytokine release and numerous organ failures, all of which contribute to poor outcomes.^[14]^ In a retrospective study of Italian patients diagnosed with COVID-19, the clinical impact of hyponatremia and its association with IL-6 levels was investigated.^[14]^ Upon admission, over 50% of the patients had low serum sodium concentrations. Furthermore, the same study demonstrated that patients with hyponatremia had worse outcomes than those without hyponatremia.^[14]^

#### 3.2.4. Heart damage by SARS-CoV-2 infection and hyponatremia.

Cardiac muscle damage with high serum troponin levels has been reported.^[41]^ The most likely mechanisms for heart injury appear due to direct myocardial damage caused by SARS-CoV-2 cardiomyocyte invasion, in addition to the systemic inflammation effects on the heart,^[42]^ leading to muscle hypofunction and heart failure.^[43]^ Heart failure can cause hypervolemia and hyponatremia.

Myocytes have ACE2 on the cell member, acting as a viral gateway and facilitating SARS-CoV-2 entrance into the myocytes, manifesting with different cardiac events.^[44]^ This study adds to the growing evidence linking hyponatremia and SIADH in COVID-19 patients. According to the authors, nonosmotic stimulation of ADH secretion and SIADH development could be caused by steroids, positive pressure breathing, and antibiotic use.^[44]^

#### 3.2.5. Lung damage.

Hyponatremia was identified in almost 35% of patients with pneumonia.^[13]^ Hyponatremia in patients with pneumonia has been linked with a higher mortality rate, signifying the need for prompt diagnosis and suitable management to improve the outcome.^[13]^ Infection with COVID-19 causes various degrees of lung involvement, ranging from mild to severe lung damage. The necessity for oxygen, steroids, and the likelihood of employing mechanical ventilation increases as lung involvement becomes more severe. This increases the risk of hyponatremia in COVID-19 by causing more lung injury, producing more ADH, and inducing SIADH.

In HIV, hyponatremia might be due to SIADH, volume depletion due to cortisol deficiency, or gastrointestinal fluid loss. *Pneumocystis carinii*, or other organisms infecting the lungs and/or CNS opportunistic infections, which also cause SIADH,^[45]^ by increasing ADH or ADH-like protein production. The same approach can be applied to COVID-19.

#### 3.2.6. Intestine damage.

A report noted that approximately 60% of patients with COVID-19 who developed watery diarrhea had moderately low serum sodium levels. It has been claimed that hyponatremia may be secondary to viral replication in intestinal epithelial cells.^[21]^ Patients with a serum sodium level of 120 mmol/L were reported to have a positive SARS-CoV-2 infection. Although the underlying hyponatremia mechanism was not clear, the reporters linked the cause of hyponatremia to SIADH and sodium loss in the watery stool.^[24]^

### 3.3. Hyponatremia versus hypernatremia in SARS-CoV-2 infection

Hyponatremia is linked with a higher proclivity of fever and nausea in SARS-CoV-2 infected individuals, with leukocytosis, neutrophilia, and increased C-reactive protein levels. Hypernatremia was less common in COVID-19 patients than in hyponatremia (2.4% vs 9.9%).^[43]^ Clinical diseases and biological anomalies were the only disparities between patients with hypernatremia and those with normal serum sodium levels.^[40]^ There was no significant difference in the length of hospitalization between the hypernatremia and normal-natremia groups, but patients with hyponatremia required prolonged hospital stay.^[43]^ It was observed that hyponatremia might be a factor that indicates a poor prognosis in a study of 323 patients with COVID-19 and longer hospital stay compared with normal serum sodium COVID-19 patients (34% vs 14%.^[46,47]^

### 3.4. Presentation and clinical evaluation of COVID-19 patients

The clinical presentation of COVID-19 patients varies and is commonly unpredictable, and these patients may have no symptoms or may present with severe complications. In addition to the usual prodromal presentations of viral infections, COVID-19 patients may present with ARDS and embolic manifestations, such as pulmonary embolism. Cardiac involvement manifests with different cardiac arrhythmias, acute pericarditis, myocarditis, cardiomyopathy, heart failure, and shock. Brain involvement is not uncommon; acute encephalomyelitis, acute hemorrhagic necrotizing encephalopathy, myoclonic seizures, acute encephalopathy, generalized myoclonus, Guillain-Barré syndrome, meningoencephalitis, and posterior reversible encephalopathy syndrome occur.^[48]^ Inflammatory auto-antibody-mediated manifestations such as Kawasaki disease, toxic shock syndrome, and secondary bacterial/fungal coinfections are common in COVID-19.

Diagnosis and illustration of hyponatremia etiology can be demonstrated by careful history and clinical examination. However, serum and urine sodium, serum and urine osmolality, thyroid function, and serum cortisol must be checked to exclude other secondary causes of low serum sodium.^[11]^ SIADH can be caused by infectious and noninfectious inflammatory disorders, malignancies, cardiovascular or hepatic diseases, ARDS, etc,^[39,49]^ which need to be excluded first.

Chest radiography is mostly performed in patients presenting with respiratory system complaints, including COVID-19 patients. Although there is a computed tomography scan grading system for lung involvement, and some studies related these grades to the degree of hyponatremia, there is no reported research to assess the relationship between chest X-ray changes and hyponatremia severity, and the correlation of these X-ray abnormalities with the outcome of COVID-19 patients. Future research projects are required to investigate these issues further.

### 3.5. COVID-19-associate hyponatremia management

There are no clear clinically universal guidelines for the management of hyponatremia in COVID-19. The treatment of electrolyte abnormalities and body fluid disturbances is guided by etiology, patient volume status, and associated comorbidities. The rate of corrected serum sodium of <10 mmol/day is almost certainly the best rate of correction for most hyponatremia-related SIADH patients.^[50]^

When the patient is overloaded, fluid restriction and electrolyte management at a slow controlled rate is an advisable primer therapy. Broadly, the available guidelines command the initiation of electrolyte correction therapy in hypovolemic hyponatremia, especially in gastrointestinal fluid loss, low salt intake, excessive fluid intake, and vigorous diuretic therapy.^[13]^ In SIADH syndrome, fluid restriction is mandatory, and some patients may need hypertonic saline infusion when they have severe neurological symptoms or signs. These treatment regimens must be conducted carefully to prevent iatrogenic pulmonary edema and lung injury aggravation due to SARS-CoV-2 infection.^[13]^

## 4. Conclusions

SARS-COV-2 patients may present with symptomatic or asymptomatic hyponatremia. In moderate to severe hyponatremia there might be neurological features, making early detection of COVID-19 infection difficult; so physician should be aware of the atypical presentations. There are several causes of hyponatremia during the recent COVID-19 epidemic, and the underlying mechanisms are not well established. Therefore, the management plan for hyponatremia should be individualized. The crucial point is that other Before diagnosing SAIDH as the cause of hyponatremia in COVID-19, alternative sources of hyponatremia should looked.

**Figure 2.**
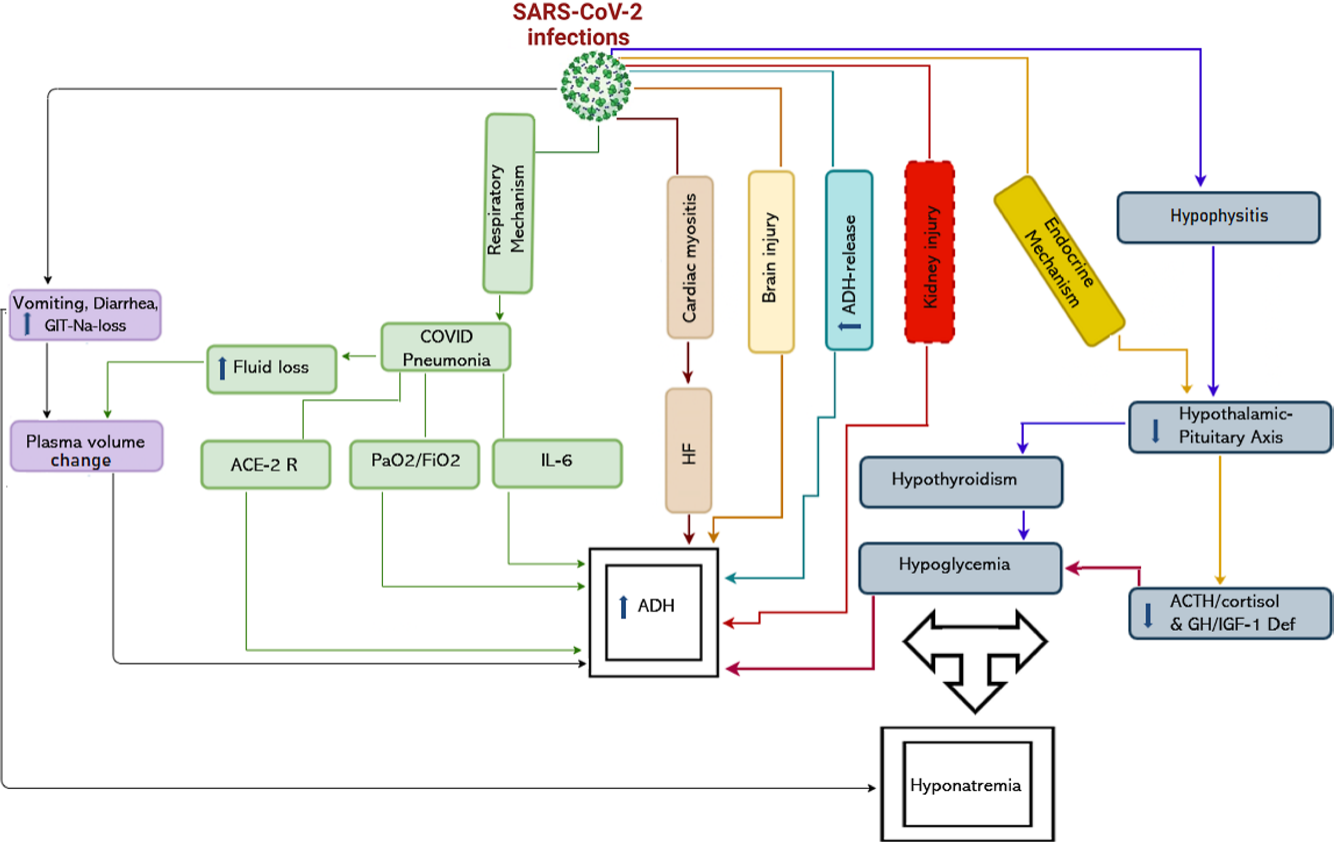
The figure shows the pathogenesis of hyponatremia in COVID 19 patient.

## Acknowledgments

I wish to express my gratitude to the Internal Medicine Residency Program and the Qatar National Library for scientific support.

## Authors contributions

Aml Habas: writing editing final approval

Elrazi Ali: writing editing final approval

Elmukhtar Habas: writing editing final approval

Amnna Rayani: writing editing final approval

Hafedh Ghazouani: writing editing final approval

Fahmi Khan: writing editing final approval

Khalifa Farfar: writing editing final approval

Abdel-Nasser Elzouki: writing editing final approval
